# Ensuring patient safety during cystocopy: risk assessment of device reprocessing through healthcare failure mode and effects analysis

**DOI:** 10.1007/s00345-025-05617-1

**Published:** 2025-04-30

**Authors:** Miriam Roncal Redin, Cristina Diaz-Agero Perez, Cornelia Bischofberger Valdes, Diego San Jose-Saras, Paloma Moreno-Nunez, Jorge Vicente-Guijarro, Jesús M. Aranaz-Andres

**Affiliations:** 1 Hospital Universitario Ramón y Cajal, Ctra. de Colmenar Viejo, km. 9,100, 28034 Madrid, Spain; 2 Instituto Ramón y Cajal de Investigación (IRYCIS), Madrid, Spain; 3 International University of La Rioja, Logroño, Spain; 4 Centro de Investigación Biomédica en Red de España (CIBERESP), Madrid, Spain; 5 University of Alcalá (University of Alcalá), Faculty of Medicine, Department of Medicine and Medical Specialties, Alcalá de Henares, Spain

**Keywords:** Patient safety, Cystoscope, Sterilization, Infection prevention, Failure mode and effects analysis, Risk analysis

## Abstract

**Introduction:**

Flexible cystoscopy is a widely performed urological procedure, with millions conducted annually. However, cystoscope contamination rates are relatively high, ranging from 2 to 21%, and infections may go undetected. Proper reprocessing of cystoscopes is a critical step in ensuring the safe use of these devices, which underscores the importance of studying it more thoroughly. The aim of this article is to describe the stages of cystoscope reprocessing in the Urology Department of a tertiary Hospital, identifying the potential failures of the system before they occur, quantifying its risks and recommending improvement actions to enhance overall safety and efficiency.

**Material and methods:**

This qualitative study was conducted using the Failure Mode and Effects Analysis methodology in different phases. First, data on structure, equipment and work processes were collected through direct observation and staff interviews. Then, processes and sub-processes were described along with their possible failures and underlying causes. Risks of this potential failures (failure modes) were quantified using the Risk Priority Number (RPN), calculated as the product of their frequency, severity and detectability. Improvement actions to mitigate failures with the highest NRP were proposed based on expert consensus and literature review.

**Key findings and limitations:**

9 processes and 11 sub-processes were identified, along with 16 failure modes. The key failure modes, those with an RPN > 100 included incorrect manual high-level disinfection, incorrect cleaning due to lack of brushing and lack of traceability. These failures were primarily caused by insufficient staff training, short intervals between procedures, and inadequate infrastructure. To address these issues, expert recommendations included providing comprehensive training for staff, improving reprocessing infrastructure by optimizing room layout, and ensuring an adequate supply of cystoscopes to reduce time constraints. Additionally, prioritizing automatic high-level disinfection and implementing a traceability system were proposed to enhance process reliability and patient safety.

**Conclusion:**

Inadequate organizational structure and equipment can compromise patient safety during high-care activities. After analyzing cystoscope reprocessing at our center, three critical failure modes (NPR > 100) were identified: lack of traceability, improper cleaning due to insufficient brushing, and improper manual high-level disinfection (HLD). To address these issues, we proposed actions including improve staff training, increase the cystoscope supply, and enhance the unit’s infrastructure. These findings may guide other healthcare professionals in improving reprocessing safety.

## Introduction

Flexible cystoscopy is one of the most commonly performed urological procedures, with nearly 4 million cystoscopies conducted annually in Europe and over 3.5 million in the United States [1]. Cystoscopes allow for the direct visualization of the lower urinary tract, primarily serving as a diagnostic tool, though they are also used for various therapeutic procedures. These are complex instruments and are classified as semicritical devices according to the Spaulding classification, as they come into contact with mucous membranes [2]. This implies the need for at least high-level disinfection (HLD) between patients. HLD can be performed manually or automatically using washer disinfectors that comply with UNE-EN ISO 15883 standards.

During cystoscopy, microorganisms can be transferred from the urethra to the bladder, potentially leading to urinary tract infections (UTIs). The reported incidence of infection following flexible cystoscopy varies widely, ranging from 2% to 21.2% according to different studies [3–6]. This incidence is particularly relevant since UTI symptoms can be easily mistaken for the usual discomfort following the cystoscopy procedure. In one study involving over 3,108 cystoscopies, 22% of patients developed asymptomatic bacteriuria post-procedure, while 59 patients (1.9%) had a clinical UTI [7]. Inadequate reprocessing of cystoscopes significantly increases risks to patient safety. Reusable endoscopes, in particular, have been strongly associated with higher rates of infection and outbreaks compared to other medical devices [8, 9] and microbial surveillance of these devices has revealed contamination rates of over 30% [10]. At our centre, recent microbial surveillance control found that 12.5% of cystoscopes were contaminated.

Cystoscope reprocessing involves a series of steps, beginning with pre-cleaning in the examination room immediately after the procedure to remove and loosen debris, preparing the instrument for subsequent manual cleaning. The cystoscope is then transferred to the reprocessing area, where it undergoes thorough cleaning, including full immersion in enzymatic detergent, brushing and flushing of all channels and ports. Manual cleaning is considered the most critical step in the reprocessing workflow, as residual bioburden can significantly compromise the effectiveness of both HLD and sterilization. Following manual cleaning, the cystoscope undergoes HLD (performed either manually or automatically) followed by meticulous rinsing, preferably with filtered water, and drying. Once reprocessed, the endoscopes should be stored in an upright position in a dry, ventilated area of sufficient size to prevent recontamination and to protect the equipment from potential damage [11]. All steps in the reprocessing cycle should be documented to ensure quality assurance and enable patient traceability if needed [12]. Additionally, a leak test and an inspection of the cystoscope's integrity should be conducted at every stage of the process to ensure the device remains safe and functional.

Numerous guidelines and recommendations [11–13] for the reprocessing of endoscopes have been published, yet compliance with these standards remains consistently low [14, 15]. Given that, the challenges in detecting post-procedural infections and the high volume of cystoscopies performed daily, mitigating patient risks is imperative. Healthcare Failure Modes and Effects Analysis (HFME), offers a valuable approach for prospectively evaluate the reprocessing cycle of these devices. This methodology focuses on identifying and addressing latent system failures before they lead to adverse events (AEs) [16], analysing their possible causes, and propose solutions to prevent harm. Therefore, our aim is to describe the cystoscope reprocessing steps within the Functional Testing Unit of the Urology Department at a tertiary Hospital, to identify potential critical system failures that contribute to an increased risk for the patient and quantify their associated risks. The second objective was to propose improvement actions to eliminate or mitigate these risks effectively.

## Materials and methods

A qualitative study was conducted following the Failure Modes and Effects Analysis (FMEA) methodology. FMEA is a proactive risk management tool for identifying the possible failure of a system, process, product or service, analysing the causes and effects of this failures, and eliminating or reducing the most significant ones by proposing risk mitigation actions [16]. It involves creating a detailed, step-by-step description of the process under review, identifying potential failures (failure mode) at each stage, and assessing their potential impact on patient safety [17]. Each identified failure is evaluated based on its frequency, severity and detectability, allowing for targeted interventions to minimize risk and enhance process reliability.

### The study was carried out in different phases

In the first phase, we defined the process to be analysed (i.e., cystoscopes reprocessing in the Urology Department) and formed a multidisciplinary team. The team members were carefully chosen to represent all key stakeholders involved in the cystoscope reprocessing workflow. It included two physicians and one medical resident from the Department of Preventive Medicine and Public Health, the nurse assistant responsible for daily reprocessing of the cystoscopes, and the head nurse of the Urology Department.

In the second phase, data on the structure, activities, equipment and procedural steps to be evaluated were collected through direct observation and staff interviews. This involved two visits per week for approximately two hours each, over the course of one month. The cystoscope reprocessing processes and sub-processes were then outlined, and a flowchart was created to identify the areas requiring focus.

Finally, possible failure associated with each subprocess were documented, and through team consensus, the causes of each failure were analysed, along with their potential impact on patient safety. Subsequently, the risk of each identified potential failure was quantified by calculating the Risk Priority Number (RNP). The RNP is used to establish a hierarchy and prioritize failures by multiplying the frequency (F), severity (S) and detectability (D) of each failure.

The failures with the highest RNP scores were prioritized to select the corresponding improvement actions. These actions were determined by a panel of experts from the preventive medicine and public health service, based on their experience and the literature reviewed. At the end of the process, a comprehensive report was generated, detailing the methodology, results, and recommended improvements, based on the causes most strongly associated with multiple failure modes.

## Results

**Fig. 1:**
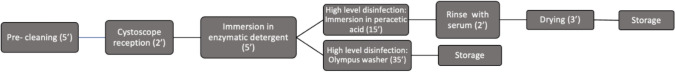
Reprocessing flowchart

After conducting the visits to the unit, we observed that the Functional Testing Unit of the Urology Department at Ramón y Cajal Hospital performs an average of 28 cystoscopies per day, ranging from a minimum of 16 to a maximum of 40, 85% of procedures involving outpatients. The most common indications for cystoscopy include bladder tumours, haematuria, ureteral catheter removal, and the assessment and treatment of urinary incontinence. The unit is equipped with 4 flexible cystoscopes and 1 rigid cystoscope, all of which are reprocessed in a dedicated area within the unit. This area is equipped with the necessary products, a washbasin and two Olympus washer-disinfectors (mini-ETD 2). Reprocessing is carried out either manually or automatically. In the manual reprocessing cycle, visible debris are first removed, followed by brushing and cleaning of the channels. The cystoscope is then immersed in an enzymatic detergent for 5 min, rinsed, and immersed in a high-level disinfectant (peracetic acid) for 15 min. The cystoscope is then rinsed with sterile water for an additional 5 min. Finally, the cystoscope is dried and stored. For automatic reprocessing, visible debris are removed, and the channels are brushed. The cystoscope is then immersed in enzymatic detergent for 5 min, followed by a HLD wash with peracetic acid for 35 min, which includes rinsing and drying. Figure 1 shows the cystoscope reprocessing flowchart with the description of the process carried out after the unit visits. Given the high volume of procedures performed daily (ranging from 16 to 40) and the limited number of available cystoscopes (4 to 5), the turnover of cystoscopes is rapid, with each cystoscope being reprocessed approximately 5 times per day. Cystoscopies are scheduled every 5 or 10 min in two procedure rooms, depending on the specific procedure. The procedures usually last between 3 and 5 min, although some may extend up to 20 min. There is no designated time allocated for reprocessing between procedures, so cleaning and disinfection are usually performed manually. Automatic disinfection is reserved for the final use of each cystoscope at the end of the workday. This is due to the fact that manual reprocessing takes approximately 30 min, while automatic HLD requires approximately 45 min.

In the third phase of the study, Table 1 was elaborated. Table 1 summarizes the processes and sub-processes involved in the reprocessing of cystoscopes, along with the potential failures that can occur at each stage, their causes and consequences, as well as the scores assigned to each item based on their frequency, severity, and detectability. A total of 9 processes and 11 sub-processes were identified, encompassing 16 failure modes in total. The failure modes with the highest RPN (RPN > 100) were as follows: Incorrect manual HLD (RPN = 125), improper cleaning due to lack of brushing (RPN = 100), and lack of traceability (RPN = 100). Additionally, RPN values between 60 and 80 were assigned to four other failure modes, including: lack of pre-cleaning (or drag-cleaning), failure to perform leak tests, failure to re-qualify automatic HLD equipment, and inadequate storage of cystoscopes. The most frequently identified causes of these potential failures included insufficient supervision and training of staff, short intervals between procedures and therefore lack of time for adequate reprocessing, inadequate equipment or infrastructure, and lack of adequate records to ensure traceability (Table 1). The potential effects of these failures were the deterioration or damage of cystoscopes, improper cleaning and disinfection leading to possible biofilm formation, recontamination of materials, cross-transmission of infections, and a lack of proper quality control.

**Table 1:** Processes and subprocesses of cystoscope reprocessing

Process	Subprocess	Failure modes	Causes	Efects	F	G	D	NPR
Precleaning	Drag cleaning	Not carried out	Lack of time and training	Biofilm formation	5	3	4	60
Cystoscope reception	Cystoscope reception	Not recorded	There is no work record	There is no traceability	5	4	5	**100**
					4	4	1	16
Leak test	Leak test	Not carried out	Lack of training	Cystoscope breakage	5	4	4	80
Cleaning	Immersion in an enzymatic detergent	Lack of brushing	Lack of time	Biofilm formation	5	5	4	**100**
		Lack of washing of channels and ports	Lack of space		1	2	4	8
		Lack of immersion time control	Lack of cystoscopes	Deterioration of equipment	3	3	4	36
	Rinsing	Lack of rising	Lack of training	Residues in the cystoscope channel	1	5	4	20
Visual inspection	Inspection of cystoscopes and accessories	Additional assurance of proper cleaning and correct condition of the cystoscope	Lack of training	Deterioration of equipment, Incorrect Disinfection	5	2	3	30
Disinfection	Manual high-level disinfection	Incorrect immersion time or incomplete immersion of device and accessories	Lack of cystoscopes Lack of training	Incorrect disinfection, risk of cross infection	5	5	5	**125**
		Not rising with distilled water	Lack of training	Residues in the cystoscope, risk to the patient	2	3	5	30
	Automatic high-level disinfection	There is no cycle record	Outdated equipment	Lack of control over physicochemical parameters and traceability	5	3	4	60
Drying	Drying	No air drying with gun	Lack of space Lack of dryer air gun	Deterioration of equipment, failures in the tower when plugging in the wet corrector	4	3	3	36
				Recontamination of the material	5	3	3	45
Storage	Storage	Lack of adequate space for storage	Lack of resources	Deterioration of material Recontamination	4	4	4	64
Material delivery and traceability	Process logging and traceability	The process is not recorded	No work record	No traceability or quality control	5	4	1	20

After reaching consensus on the failure modes with the highest priority (highest RPN) and the causes of these potential failures, the expert panel, based on their experience and the reviewed literature, proposed several improvement actions: 1) providing comprehensive and sistematic training to maintain knowledge and skills since lack of training was cause of the lack of leak tests, lack of rinsing, lack of visual inspection, 2) enhancing the reprocessing infrastructure by changing the room layout to guarantee a clean and safe spaces since lack of space was cause of the lack of channel and port washing, lack of drying, and inadequate storage of cystoscopes and 3) ensuring an adequate supply of cystoscopes to address time constraints, which was the cause of lack of pre-cleaning, lack of brushing, lack of immersion time control, and insufficient or incomplete immersion of the cystoscope for proper manual HLD). To calculate the appropriate number of cystoscopes, the average (28) and maximum daily number of procedures performed in the unit (40) were taken into account, as well as the time required for the correct reprocessing of the devices, including all the recommended steps (pre-cleaning, immersion in enzymatic detergent and high-level disinfection in an automatic washer-disinfector), which is 45 min. With these premises and calculating one scheduled procedure every 10 min in the two procedure rooms, lasting 5 min each, the minimum desirable number of cystoscopes available in the unit should be 8. Other recommendations, such as the prioritization of automatic HLD in washers and the implementation of a traceability system, were also suggested.

## Discussion

Our results identified key areas for improvement in three processes: in cystoscope reception, lack of traceability (RNP = 100), in the cleaning phase, inadequate immersion in enzymatic detergent and lack of brushing (RNP = 100) and in disinfection, incorrect manual HLD (RNP = 125).

This finding aligns with other studies, which emphasize that cleaning and manual disinfection are the most critical steps in cystoscope reprocessing and report the highest failure rates in these stages [9, 18, 19]. On one hand, cleaning plays a crucial role in removing all visible soil and significantly reducing the bioburden, thus facilitating the biocidal process. Both the interior and exterior of the cystoscope must be thoroughly cleaned, as this step is vital for the success of subsequent microbicidal processes used for disinfection or sterilization [11]. In our study, the lack of brushing was due to time constrains and had a significant impact, contributing to one of the highest NRP values.

On the other hand, the most frequent error, with the highest NPR, was incorrect manual HLD, resulting from either failing to fully immerse the cystoscope and its accessories in the high-level disinfectant, or from immersing them for an insufficient amount of time. The effectiveness of disinfectants is influenced by the physical condition of the endoscope (such as cracks or lumens), the microbial load and, most importantly, the concentration, water temperature and exposure time to the chemical solution. These variables are difficult to control in manual disinfection processes. Therefore, whenever feasible, automated HLD is strongly recommended. Manual decontamination methods do not ensure standardized procedures or effective removal of biofilms from cystoscope channels [20]. Moreover, automated methods eliminate the risk of staff exposure to disinfectants and the potential toxicity from direct skin contact or inhalation of vapours. A study by Ofstead et al. analysed the benefits of automated versus manual endoscope reprocessing. It found that personnel performing manual reprocessing adhered to cleaning guidelines only 1.4% of the time, compared to a compliance rate of 75.4% for those using automated washer disinfectors. The study also revealed that staff using manual methods reported significantly more health issues, such as physical injuries or symptoms related to chemical inhalation, than those who used automated disinfection [19]. Additionally, automated endoscopes reprocessing has proven to be cost-effective. In countries where productivity improvements can translate into revenue, transitioning to automated reprocessing has a positive financial impact, with capital investment being amortized within months [21]. This savings from automated endoscope reprocessing quickly offset the initial cost associated with purchasing and maintaining the equipment [20]. While automated cystoscope reprocessing does have some drawbacks, such as material compatibility issues, the risk of residual contamination, higher costs, and the potential for technical failures, it has been demonstrated to be a faster, more reliable, and safer reprocessing method [22]. At our centre, the high volume of functional tests performed, combined with their short duration—usually less than 5 min—and the limited number of available cystoscopes (5) led to a preference for manual reprocessing, as it is faster than automatic reprocessing.

Moreover, the lack of documentation for reprocessing procedures significantly impedes traceability and product control, both of which are critical for ensuring patient safety and demonstrating compliance with regulatory requirements. In fact, the implementation of a traceability system could soon become mandatory. The new EU Regulation 2017/745 on Medical Devices (MDR) emphasizes of the importance of traceability by introducing a unique device identification (UDI) system for all marketed medical devices [23]. Effective traceability systems not only enhance patient safety but also contribute to process improvement, optimized productivity, efficient device management, and the prevention of healthcare-associated infections [24–26]. In our center, the lack of traceability was due to the absence of a work log, coupled with a lack of awareness regarding the importance of traceability.

We identified several causes of failure including insufficient staff supervision and training, lack of time for reprocessing between procedures and inadequate equipment or infrastructure. Availability issues are a common concern in many centres among urologists. According to a survey conducted in 2020 in Germany, France and the UK, 19.1% of urologists report frequent problems with cystoscope availability, while 65.8% noted that they occasionally had to wait for a cystoscope to arrive before beginning an examination [1]. There are two possible solutions to increase the availability of cystoscopes without compromising the quality of their disinfection: switching to disposable cystoscopes, thereby eliminating cleaning and disinfection times, or increasing the number of reusable cystoscopes. A recent study described the experience of a centre that transitioned to 100% disposable cystoscopes, reporting similar costs and a reduction in waste and water consumption compared to reusable devices. However, the study focused on a single cystoscope model in one centre, which introduces limitations to its generalizability [28]. Regarding environmental impact, another study compared the carbon footprint of a disposable cystoscope, from manufacturing to recycling, with that of a reusable flexible endoscope undergoing standard HLD with peracetic acid. The single-use cystoscope showed a 33% reduction in the climate change category [29]. On the other hand, other articles have shown a lower carbon footprint with the use of reusable devices compared to disposable devices, as long as HLD is performed using automatic methods [30]. Finally, after evaluating the options and given that the evidence on the feasibility and environmental impact of using disposable cystoscopes is controversial, one of our proposed improvement measures was to increase the number of reusable cystoscopes available from 5 to 8. This adjustment would align with the volume of procedures performed and facilitate the use of automatic reprocessing, ensuring a more sustainable and efficient workflow.

Lack of training of the reprocessing personnel was also a critical step found. Proper training helps ensure that cleaning and disinfection procedures are performed correctly, safeguarding patient safety and maintaining the integrity of the devices. Properly trained technicians demonstrate better compliance with protocols, greater confidence in their work, and higher satisfaction levels [32]. In this regard, the brand supplying the cystoscopes was approached to provide training. Finally, the lack of adequate equipment and infrastructure in the reprocessing area was identified as another contributing factor to several failure modes. It was observed that instruments were not stored properly in a clean, dust-free and well-ventilated area, increasing the risk of cystoscope contamination during storage [31]. Therefore, improving the infrastructure of the unit was proposed to ensure a clear separation between soiled and clean workspaces, with sinks large enough to fully immerse cystoscopes and their accessories and proper storage conditions for equipment after cleaning and disinfection. These proposed changes were deemed not only feasible at the organisational level, but also highly impactful. They would significantly improve the reprocessing procedures, modernize the unit and create a solid foundation for implementing.

HFMEA is a methodology typically implemented in order to identify potential failures and assess their severity. In our study, we applied this methodology to tackle a challenge that is difficult to address with quantitative techniques: evaluating errors in the reprocessing of medical instruments [14], a problem that can lead to serious adverse events, particularly cross-infections [3]. This issue is especially significant given that cystoscopy is generally considered a low-risk procedure and is not routinely monitored. However, studies investigating infection rates and morbidity associated with cystoscopy have revealed that up to half of patients the report urinary symptoms, such as dysuria, increased urinary frequency or haematuria after the procedure, with the incidence of infection following flexible cystoscopy ranging from 2% to 21.2% [32–35]. Even at the lower end of this spectrum, the high volume of procedures performed each year would result in a substantial public health impact.

Our study has several limitations. First, the FMEA process relies on the expertise and observations of committee members to identify failures, which means that there is a possibility that some failure modes might go undetected, potentially overlooking areas for improvement. Moreover, certain aspects of FMEA, such as the concept of detectability, and scoring of risks are inherently subjective and led to some debate within the team members [37]. To address this, FMEA was used as an active tool to systematically analyse potential failures and investigate their causes. This is especially important as we do not have good systems for detecting failures once they have occurred (cystoscope sampling systems are not comprehensive, UTI symptoms are mistaken for common complaints after cystoscopy and there are no cystoscope-patient traceability systems). Therefore, the use of proactive methodologies, such as FMEA, to identify failure points and prevent the occurrence of the event are essential. Moreover, being a qualitative study, some data were collected through staff interviews, which may have introduced some subjectivity in the information. To mitigate this, the researchers conducted multiple visits to the reprocessing unit and held interviews with staff on several occasions to ensure more comprehensive and consistent data collection. Additionally, identifying risks in collaboration with the staff involved has facilitated the implementation of changes, fostering a culture of safety and collaboration between departments, and producing results tailored to the unique needs of each unit.

This study shows that the infection control team should be more present in the units where invasive procedures are performed and should not reduce its tasks to microbiological surveillance but also include visits and audits to verify staff competency and adherence to the recommended reprocessing practices and provide continuous feedback. Studies have shown that strict adherence to all steps of manual endoscope reprocessing is rare, and critical tasks are often omitted, with the risk of cystoscope contamination that this entails. Furthermore, our results coincide with those of the literature consulted, indicating that our areas for improvement are likely to be assessed by other centres. In addition, the FMEA methodology is simple and widely validated, and it can be used in any centre.

Future research should focus on evaluating the cost-effectiveness of reusable devices, considering both their upfront costs and long-term sustainability, as it would play a crucial role in optimizing healthcare practices and ensuring efficient use of resources.

## Conclusion

In our centre, after an in-depth analysis of the steps involved in cystoscope reprocessing, three high priority failure modes (NPR > 100) were identified: lack of traceability, improper cleaning due to lack of brushing, and improper manual HLD due to insufficient immersion time or incomplete immersion of the device. Several feasible improvement actions were proposed based on the causes of these failures, including improving staff training, increasing the number of available cystoscopes to 8 and improving the infrastructure of the unit. Our experience may be useful for other healthcare professionals seeking guidance in this area.

High care activity combined with insufficient organisational structure and instrumentation can compromise patient safety. It is therefore essential to ensure adequate infrastructure and equipment to perform reprocessing safely, as well as continuous surveillance to ensure cystoscope-patient traceability and staff training.

## Data Availability

No datasets were generated or analysed during the current study.
